# Chordoid glioma: a rare radiologically, histologically, and clinically mystifying lesion

**DOI:** 10.1186/s12957-015-0603-9

**Published:** 2015-05-28

**Authors:** Daniele Bongetta, Andrea Risso, Patrizia Morbini, Giorgio Butti, Paolo Gaetani

**Affiliations:** Neurosurgery, Department of Clinical-Surgical, Diagnostic and Pediatric Sciences, Università degli Studi di Pavia, Piazzale Golgi 19, 27100 Pavia, Italy; Unit of Pathology, Department of Molecular Medicine, University of Pavia and Fondazione IRCCS Policlinico S. Matteo, Piazzale Golgi 19, 27100 Pavia, Italy; Neurosurgery Unit, Fondazione IRCCS Policlinico S. Matteo, Piazzale Golgi 19, 27100 Pavia, Italy

**Keywords:** Chordoid, Chordoid glioma, Glioma, Third ventricle

## Abstract

Chordoid glioma (CG) is a rare central nervous system neoplasm (WHO grade II) of uncertain origin whose typical localization is in the anterior part of the third ventricle. Its clinical, radiological, and histological features may vary and furthermore mimic other kind of benign lesions usually associated with a better outcome. We report a case of a 43-year-old female who underwent gross total removal of a lesion of the third ventricle causing hydrocephalus. The imaging studies and the intraoperative examination led at first to a hypothesis of meningioma. Early surgical and neurological outcomes were good. The patient underwent multiple complications related to hypothalamic dysfunctions and thrombohemorragic issues and eventually died because of systemic infections. Definitive examination was of chordoid glioma of the third ventricle. Reviewing literature, we evaluated possible pitfalls in radiological and histological diagnosis as well as in surgical and medical treatment of CGs. Despite their benign presentation, a high incidence of multiple possible severe complications is reported. Early alertness and combined treatment strategies could improve overall CGs treatment strategies.

## Background

Chordoid glioma is a rare glial neoplasm firstly labeled as a new pathologic entity by Brat et al. in 1998 [1], although Wanschitz et al. described its features as a meningioma variant in 1995 [2]. World Health Organization 2007 classification assigned it a grade II [3]. It usually occurs in adults (mean age, 46) with a female predominance of 2:1; only three pediatric cases are reported in literature [4–6]. Clinical presentation is usually with headache, visual disturbances, and memory deficits [7]; less frequent presentation is with hypothalamic and/or hypophysis disturbances (amenorrhea, hypothyroidism, weight gain, polydipsia) [8]; other focal deficits are rare. Its localization is usually in the anterior part of the third ventricle with variable extension to the suprasellar region and lateral ventricles; hydrocephalus is present in nearly 1/4 of patients depending more on tumor location rather than size [9]. Radiological findings usually show a well-circumscribed ovoid lesion hyperattenuating at computed tomography (CT) scans and T1-weighted isointense and T2-weighted hyperintense at magnetic resonance imaging (MRI) scans. Contrast enhancement is vivid; there may be cystic changes and rare calcifications [10–12]. Chordoid glioma histologic features show clusters and cords of epithelioid cells with eosinophilic cytoplasm embedded in a mucinous matrix rich in lymphoplasmacellular infiltrates and Russell bodies; mitotic activity, vascular proliferation, nuclear atypia, and necrosis are rare or absent [13, 14]. Immunohistochemical analysis is characterized by strong and diffuse reaction to glial fibrillary acidic protein (GFAP) and Vimentin; epithelial membrane antigen (EMA), CD34, S-100, and cytokeratin are expressed in more than 2/3 of samples [15]. Neurofilament protein, synaptophysin, and p53 are rarely positive; MIB-1 index is always lower than 5 %. The general hypothesis of the chordoid glioma histogenesis favors a glial origin, especially from the ependymal cells called tanycytes [16], located in the anterior part of the third ventricle; a hypothesis of a multipotent stem cell of Rathke’s pouch [17] or even ependymal origin have been reported [18]. As has been recently reported, thyroid-transcription factor-1 expression may histogenetically link these lesions to other morphologically heterogeneous neoplasms in the third ventricular region [19]. We report a case with a perioperative satisfactory result, whose original misdiagnosis may have led to underestimate the incidence of possible threatening complications.

## Case presentation

**Fig. 1:**
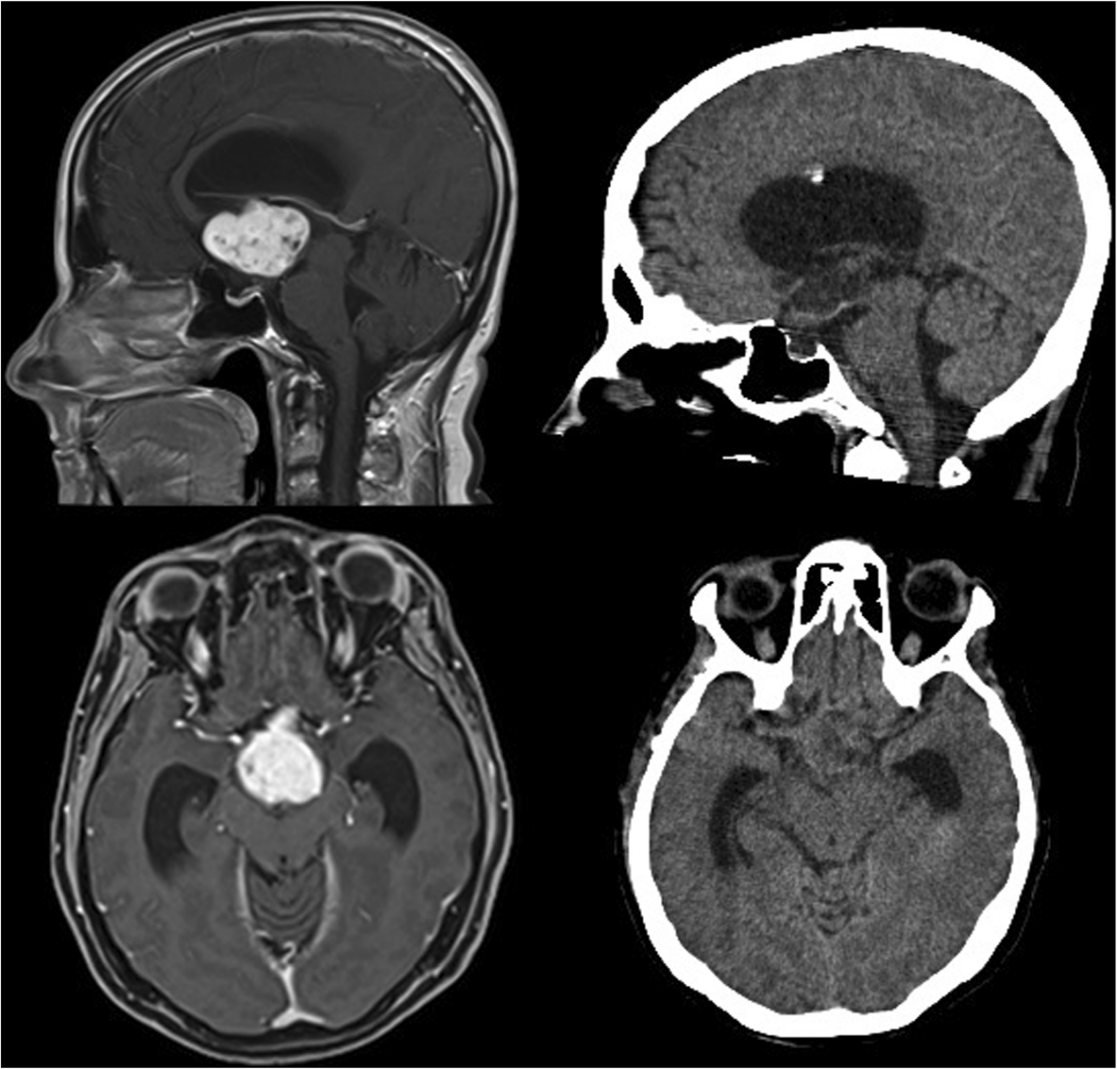
Radiological evaluation. Left: T1-weighted MRI scan showing a lesion in the medial mesencephalic region with heterogeneous contrast enhancement. Center: early postoperative CT scan showing gross total removal

We present a case of a 43-year-old woman, with a 6-year history of diabetes mellitus. In the last few weeks preceding her hospitalization she suffered from headache, asthenia, and mood depression; since her headache kept worsening, she was eventually admitted to our Hospital. Neurological examination showed no significant deficit, electroencephalogram (EEG) was normal, fundus oculi study showed an embossed, blurred papilla with peripapillary hemorrhage in the right eye, slightly blurred margins of the left eye papilla. She underwent a CT scan which showed a 4 × 3 × 3 cm lesion in the medial mesencephalic region with heterogeneous density with hyperdense peripheral ring and vivid heterogeneous contrast enhancement causing noncommunicating hydrocephalus. Neither edema, nor calcifications nor cysts were noted. A hypothesis of a neoplasm with a III ventricle origin was made. A MRI scan showed a lesion slightly T1-hypointense, T2-heterogeneally hyperintense with vivid heterogeneous contrast enhancement involving little necrosis areas, cranially dislocating the fornix, minimally proceeding into the lateral ventricles through the foramina of Monro, and anteriorly dislocating the lamina terminalis. A millimetric heterogeneous T1-hyperintense and GE-hypointense oval area resembling a calcification of the lesion margin was noted near the right cerebral peduncle (Fig. 1). A hypothesis of a III ventricle neoplasm originating from choroidal plexus versus intraventricular meningioma versus ependymoma was made. An external ventricular drain (EVD) was placed with relief of the headache, and she subsequently underwent gross total removal of the lesion via a right pterional approach. No major complications occurred during surgery, and the patient was admitted to the Intensive Care Unit for a controlled slow awakening. Neurological examination showed no significative deficit, intracranial pressure (ICP) never went above 20 mmHg. In the early days she presented tendency to hypernatremia, with hyperosmotic urine output. After the onset of fever due to *Escherichia Coli* urinary infection she became drowsy. A CT scan showed no anomalies. Hormonal routine revealed diminished thyroid-stimulating hormone (TSH) levels (0.142 mIU/L; 0.400–4.000). She presented a single partial seizure (EEG normal) after which she reverted to hyponatremia (128 mEq/l). At the end of the first week post intervention, she developed deep vein thrombosis in the right popliteal district. Therapeutic low molecular weight heparin therapy was administered; no indication of inferior vena cava filter placement was posed. The patient remained drowsy and once again presented tendency to hypernatremia. A MRI scan 20 days post-op was performed showing gross total removal of the tumor, heterogeneous density in the surgical field, small subacute ischemic lesions of the right caudate nucleus and of the left thalamus. She suffered from an acute onset of dyspnea and desaturation, so she underwent a chest CT scan showing bilateral pulmonary thickening in absence of frank signs of pulmonary embolism. The respiratory functions further worsened: a bronchoscopy with sampling isolated *Candida Albicans* and *Staphylococcus Aureus*, a percutaneous tracheostomy was performed. Respiratory and neurologic functions improved. Owing to a persistent dilatation of the ventricles, she underwent a ventricular-peritoneal shunt intervention. Postoperative course was complicated by intraventricular hemorrhage and subsequent shunt occlusion (Fig. 1). The shunting was thus reverted to external with slow, progressive, neurological improvement. A new septic state with isolation of *Pseudomonas Aeruginosa* and *Klebsiella Pneumoniae* multidrug resistance (MDR) was treated with intense antimicrobial therapy. Neurological examination showed order execution with mild right side hemiplegia. She suffered again from dyspnea and progressive desaturation so she underwent a chest CT scan showing massive acute respiratory distress syndrome (ARDS) secondary to *P. Aeruginosa* and *K. Pneumoniae* MDR pneumonia which eventually led her to death about 3 months after admission.

### Histology

**Fig. 2:**
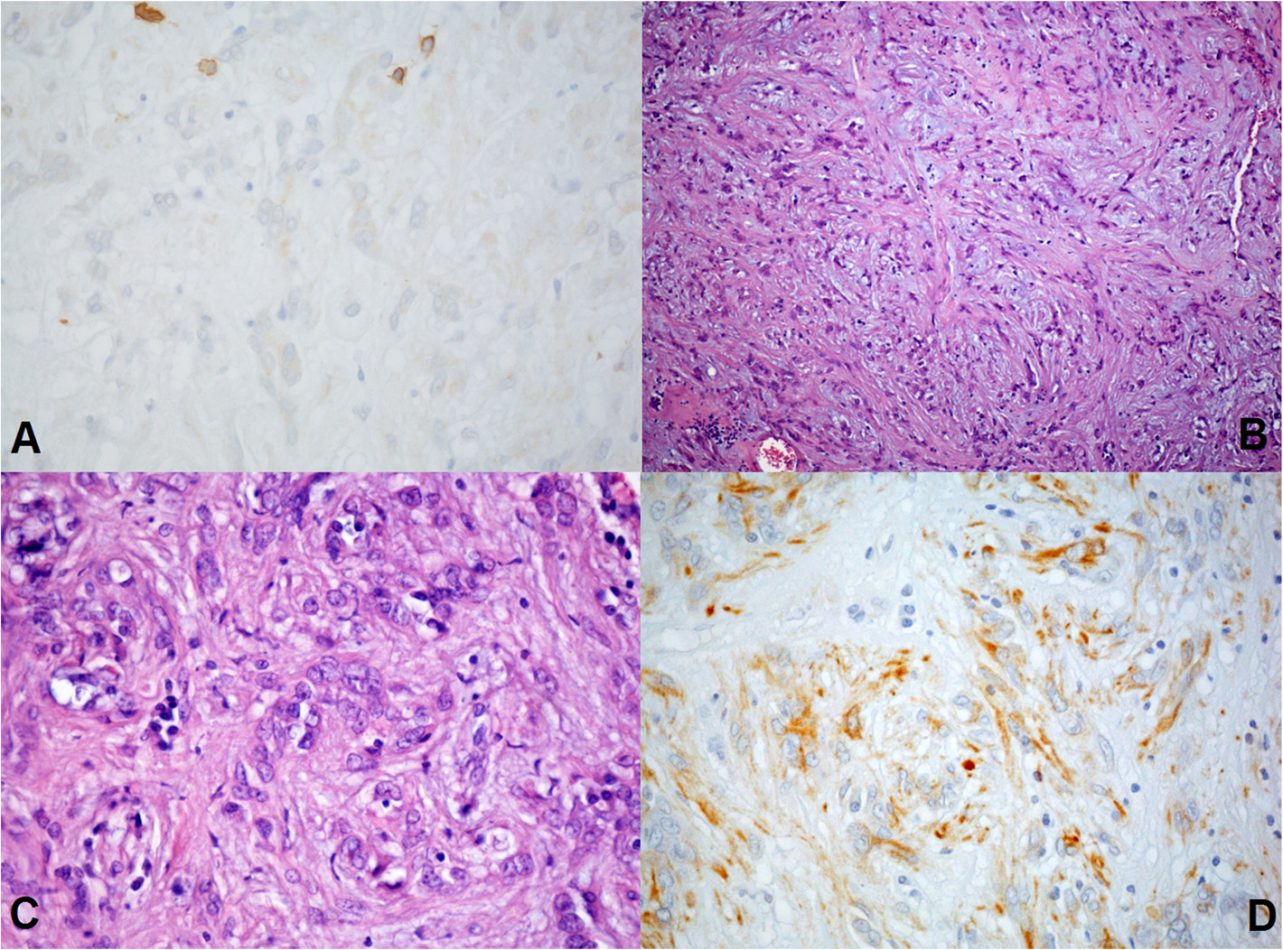
Histological evaluation. Paraffin-embedded samples of chordoid glioma documenting: **a** No immunoreactivity for EMA; **b** Epithelioid elements with regular nuclei arranged in small nests and chains immersed in abundant myxoid stroma (H-E x 10); **c** (H-E x 20); **d** GFAP

Intraoperative examination showed fragments consisting of edematous stroma, including aggregates of medium size cells with eosinophilic cytoplasm and slight nuclear pleomorphism, with no mitotic activity, nor necrosis. A provisional diagnosis of meningioma was made. The examination of formalin-fixed and paraffin-embedded samples documented a neoplastic proliferation of epithelioid elements with regular nuclei, without prominent nucleoli, arranged in small nests and chains immersed in abundant myxoid stroma. A consistent lymphoplasmacytic inflammatory infiltrate was associated, no mitosis was detected; MIB-1 proliferative index was of about 3 %. The neoplastic elements were intensely immunoreactive for Vimentin and GFAP and moderately to S-100. No immunoreactivity for cytokeratin, EMA, or progesterone receptor was observed. Chordoid meningioma, chordoma, and chordaoid glioma of the third ventricle were considered in the differential diagnosis. The absence of both a typical meningothelial proliferation and EMA and progesterone receptor expression allowed us to discard the hypothesis of chordoid meningioma. The diagnosis of Chordoma was considered unlikely on account of the immunohistochemical profile and neoplastic cells morphology. The location of the lesion as well as its histological characteristics and antigenic pattern led to a diagnosis of chordoid glioma of the third ventricle (Fig. 2).

## Discussion

Chordoid glioma is a rare central nervous system neoplasm whose determination as a distinct histological entity is relatively recent. Perioperative identification of the lesion with radiological and histological intraoperative examinations may be difficult, even by the most experienced diagnosticians. On the radiological side, common misdiagnosis may be avoided by a differential diagnostic approach based both on the typical localization of the lesion and the peculiar pattern of its growth. As a matter of fact, chordoid gliomas typically show a rather common benign contrast enhancement pattern. Different MRI sequences do not show lesion-specific characteristics; hypothesis of different frequent low grade neoplasm may be wrongly made. In particular, meningiomas are more commonly related to a skull base dural feeding, and their growth pattern is typically oriented to the sellar region. Moreover, ependimomas and furthermost choroidal plexus neoplasms are rarely located in the anterior midline. Ectopic pituitary adenomas are anecdotal. We agree with Smith et al. in stating that location, age, gender, and underlying conditions may help narrow the differential diagnosis [12]. A widespread knowledge of this newly defined third ventricle pathological entity in the radiologists’ community appears to be mandatory. As for histological examination, recent publications report that intraoperative smear cytology could reveal distinctive cytological features, identifying the unique histological pattern of chordoid gliomas [14, 20]. In literature, we found that the main problem lied in telling a chordoid glioma from a chordoid meningioma. As for the radiological issues, a topographic approach could favor a correct diagnosis as early as at an intraoperative analysis stage. Postoperative outcome may widely vary from a no-deficit patient to a relatively high death rate in the first month. Surgical interventions are often reported as relatively easy and uneventful, suggesting that many variables and small details are to be taken into account in the outcome analysis. Recent reviews show a postoperative mortality up to 17 % while deaths related to pulmonary embolism and infectious diseases account for 45 and 36 %, respectively [21]. Diabetes insipidus and hypothalamic disorders are common complications too. The series of events that led to death in the case presented here may suggest that many factors may cooperate in breaking the fragile postoperative systemic equilibrium of these patients. In particular, we stress the possible combined role of multiple thrombotic risk factors: the hypothalamic disorders, the neurological deficits with prolonged sedation and ICU stays, and the histology of the glial series. The overall risk of deep vein thrombosis and related pulmonary embolism may be underestimated. We thus recommend an effective, timely antithrombotic prophylaxis. Hydrocephalus treatment must also be carefully planned considering the anticoagulation therapies. On a pure surgical level, chordoid glioma management has been described as a mere biopsy in only seven patients [8, 9, 15, 16, 22, 23]. The surgical approaches employed to access the anterior part of the third ventricle may be summoned in transventricular (transcortical or transcallosal) and trans-lamina terminalis (pterional or subfrontal route), whereas transsphenoidal sublabial was employed only once [17]. Ventricular shunting is performed as needed. No recurrence occurred after gross total removal, while 5 recurrences out of 18 available partial removal cases were reported [1, 24, 25]. Still, morbidity and mortality data progressively increase from 0 % of biopsy cases up to about 70 and 30 % in GTR cases. In our case, we chose a right pterional approach with extensive basal temporal bone drilling and performed a GTR. We evaluated that this approach could warrant an adequate exposure of the chiasmal region with a less invasive parenchymal approach compared to the transventricular and lesser frontal brain retraction compared to the subfrontal route. Literature data may suggest that the trans-lamina terminalis approach is better in terms of morbidity and mortality [21]. The choice of a surgical route may be mainly due to the presence of objective variables such as ventricular extension and laterality and also to the subjective surgeon’s expertise and confidence. Even the intraoperative diagnosis plays a role in determining the extension of the resection. The typical misdiagnosis of meningioma at smear analysis, as in our case, could favor a more exhaustive removal approach. There are also reports of several other adjuvant therapies such as conventional radiotherapy, stereotactic radiosurgery, intratumoral radiotherapy, and Gamma Knife Surgery [1, 15, 23, 24, 26–29]; no chemotherapy treatment is reported. The knowledge of these promising tools for a combined treatment approach to these lesions could modify the surgical strategy.

## Conclusions

Since most physicians have little knowledge of this relative new central nervous system tumor entity, as we had ourselves before this case study, chordoid glioma may represent a mystifying lesion in radiological, histological, and clinical terms. Extensive knowledge of diagnostic pitfalls and high incidence of multiple possible severe complications could improve the outcome of chordoid gliomas (CGs) patients. Further studies are needed to assess the correct treatment strategies in terms of complication prevention, surgical approach, and role of adjuvant therapy.

## Consent

Written informed consent was obtained from the patients for publication of this case report and any accompanying images. A copy of the written consent is available for review by the editor-in-chief of this journal

## Abbreviations

CG: chordoid glioma
MRI: magnetic resonance imaging
CT: computed tomography
WHO: World Health Organization
GFAP: glial fibrillary acidic protein
EMA: epithelial membrane antigen
EVD: external ventricular drain
ICP: intracranial pressure
TSH: thyroid-stimulating hormone
EEG: electroencephalography
ARDS: acute respiratory distress syndrome
MDR: multidrug resistance
